# Whole-genome sequence assembly of *Pediococcus pentosaceus* LI05 (CGMCC 7049) from the human gastrointestinal tract and comparative analysis with representative sequences from three food-borne strains

**DOI:** 10.1186/s13099-014-0036-y

**Published:** 2014-08-30

**Authors:** Long-Xian Lv, Yu-Dong Li, Xin-Jun Hu, Hai-Yan Shi, Lan-Juan Li

**Affiliations:** 1 State Key Laboratory for Diagnosis and Treatment of Infectious Disease, The First Affiliated Hospital, Zhejiang University, Hangzhou 310003, PR China; 2 Food Safety Key Lab of Zhejiang Province, Zhejiang Gongshang University, Hangzhou, PR China; 3 Collaborative Innovation Center for Diagnosis and Treatment of Infectious Diseases, Hangzhou, China

## Abstract

**Background:**

Strains of *Pediococcus pentosaceus* from food and the human gastrointestinal tract have been widely identified, and some have been reported to reduce inflammation, encephalopathy, obesity and fatty liver in animals. In this study, we sequenced the whole genome of *P. pentosaceus* LI05 (CGMCC 7049), which was isolated from the fecal samples of healthy volunteers, and determined its ability to reduce acute liver injury. No other genomic information for gut-borne *P. pentosaceus* is currently available in the public domain.

**Results:**

We obtained the draft genome of *P. pentosaceus* LI05, which was 1,751,578 bp in size and possessed a mean G + C content of 37.3%. This genome encoded an abundance of proteins that were protective against acids, bile salts, heat, oxidative stresses, enterocin A, arsenate and universal stresses. Important adhesion proteins were also encoded by the genome. Additionally, *P. pentosaceus* LI05 genes encoded proteins associated with the biosynthesis of not only three antimicrobials, including prebacteriocin, lysin and colicin V, but also vitamins and functional amino acids, such as riboflavin, folate, biotin, thiamine and gamma-aminobutyrate. A comparison of *P. pentosaceus* LI05 with all known genomes of food-borne *P. pentosaceus* strains (ATCC 25745, SL4 and IE-3) revealed that it possessed four novel exopolysaccharide biosynthesis proteins, additional putative environmental stress tolerance proteins and phage-related proteins.

**Conclusions:**

This work demonstrated the probiotic properties of *P. pentosaceus* LI05 from the gut and the three other food-borne *P. pentosaceus strains* through genomic analyses. We have revealed the major genomic differences between these strains, providing a framework for understanding the probiotic effects of strain LI05, which exhibits unique physiological and metabolic properties.

## Background

The genus *Pediococcus* belongs to the family *Lactobacillaceae* in the order *Lactobacillales*. Currently, it is comprised of eleven valid published species, including *Pediococcus acidilactici, P. stilesii*, *P. pentosaceus*, *P. siamensis*, *P. cellicola*, *P. argentinicus*, *P. parvulus*, *P. ethanolidurans*, *P. claussenii*, *P. inopinatus* and *P. damnosus*[1]. The majority of the members of the genus *Pediococcus* are used in the food and drink industry as starter and probiotic cultures as well as food spoilers [2]. *P. pentosaceus* has been intensively investigated and widely employed for food preservation due to its ability to produce antimicrobial agents [3]. Additionally, several strains of *P. pentosaceus* have been shown to reduce inflammation, encephalopathy [4], obesity and fatty liver [5] in animals. Although food is the main source of *P. pentosaceus* for humans, the strains of *P. pentosaceus* adapted to the gastrointestinal tract are dissimilar from those found in food because the former may originate from sub-populations present in food at low numbers that exhibit special adaptive properties [6].

Previously*,* we have isolated a potential probiotic, *P. pentosaceus* LI05 (CGMCC 7049), from the fecal samples of healthy volunteers. This strain is tolerant to bile and acid and possesses strong antimicrobial activities against tested enteropathogens. More importantly, the administration of *P. pentosaceus* LI05 during acute D-galactosamine-induced liver injury in rats was shown to reduce elevated alanine aminotransferase and aspartate aminotransferase levels, prevent the increase of total bilirubin, reduce the histological abnormalities of both the liver and terminal ileum, decrease bacterial translocation, increase the serum levels of IL-10 and result in a cecal microbiome that differ from that of the liver injury control [7].

In this study, we present a summary, classification and the unique characteristics of human gut-borne *P. pentosaceus* LI05 in addition to a high-quality draft genome sequence and annotations. The probiotic properties of *P. pentosaceus* LI05 were analyzed using these genomic sequences combined with data from our previous study. Because the genome sequences of *P. pentosaceus* SL4 from kimchi [8], *P. pentosaceus* IE-3 from a dairy effluent sample [9], and *P. pentosaceus* ATCC25745 from plant [10] are now available, this research will provide an essential resource for elucidating the differences between strains isolated from food and the human gastrointestinal tract.

## Methods

### Determination of cultural, morphological and physiological properties

Growth was investigated under different temperature, pH and NaCl conditions. Cell morphologies, motilities and sporulation activities were examined using transmission electron (H-600, Hitachi Ltd., Tokyo, Japan) microscopy. Phenotypic identification was achieved with API CH50 strips and the API CHL medium system according to the manufacturer’s instructions (BioMérieux SA, Marcy-l’Etoile, France). Other physiological and biochemical tests were conducted as described previously [11]. Phylogenetic analysis was conducted using the neighbor-joining method based on the 16S rRNA and housekeeping gene sequences [12].

### Cultural conditions and DNA isolation

After revival using standard methods, the *P. pentosaceus* LI05 strain (CGMCC 7049) was anaerobically cultured in DeMan-Rogosa-Sharpe (MRS; OXOID, Thermo Fisher Biochemicals Ltd., Beijing, China) broth at 37°C for 24 h. Cells were obtained by centrifugation at 8,000 g for 10 min at 4°C. DNA was extracted using the QIAamp DNA Micro Kit according to manufacturer’s guidelines (Qiagen, Westburgb.v., Leusden, The Netherlands).

### Genome sequencing and assembly

The genome of *P. pentosaceus* LI05 was sequenced with the next-generation sequencing platform Illumina HiSeq 2000, and the total number of reads based on a 500-bp library database were 2 × 11,079,017 (bp). The quality of the sequencing read data was estimated by calculating the quality and GC content of each read. The draft genome sequence was assembled using SOAPdenovo2 [13], and iterative optimization was used to obtain the optimal k-mer value through the use of 31–85 k-mers. The 500-bp libraries were used to build scaffolds, and the SOAPdenovo gap closer software was also used (http://soap.genomics.org.cn/soapdenovo.html). To close the remaining gaps, reference-guided assemblies were carried out with the CLC Genomics Workbench v. 6.05 (CLC bio, Aarbus, Denmark). The combination of *de novo* assembly and reference-guided assembly was performed manually using the microbial genome-finishing module in the CLC genomics workbench (CLC bio, Aarbus, Denmark). The complete genome sequence of *P. pentosaceus* ATCC 25745 was used as the reference genome.

### Genome annotation

*P. pentosaceus* LI05 genes were identified using Glimmer [14] together with comparative gene prediction by the direct mapping of the ORFs of the *P. pentosaceus* ATCC reference strain from the NCBI Genome Database. After a round of manual curation, the unannotated predicted coding sequences (CDS) were translated into amino acid sequences for a query using the NCBI non-redundant database as well as the UniProt, Pfam, COG, and InterPro databases to identify the closest existing homology annotations. Transfer RNA (tRNA) genes were detected using tRNAScanSE [15]. Ribosomal RNAs (rRNAs) were identified using a BLASTn [16] search against the ribosomal RNA databases. Signal peptides were predicted using SignalP 4.0 [17], whereas transmembrane helices in proteins were predicted using TMHMM [18]. The Integrated Microbial Genomes (IMG) platform (http://img.jgi.doe.gov/) was used to support additional gene prediction analyses and manual functional annotations [19].

### Comparative genomics

A comparative genomic analysis using BRIG [20] was conducted comparing *P. pentosaceus* LI05 from the human gastrointestinal tract with three food-borne strains with available genomic sequences, including *P. pentosaceus* ATCC 25745, SL4 and IE-3. The *P. pentosaceu*s LI05 genome sequences sharing low identities (<50%) with the other strains were designated as the *P. pentosaceus* LI05-unique regions. The proteins encoded by the genes that only existed in *P. pentosaceus* LI05 or that possessed sequence similarities of less than 50% with the three food-borne strains were further analyzed by BLASTp.

## Results and discussion

### Classification and unique features

*P. pentosaceus* LI05 is a Gram-negative, non-motile, acid-tolerant, non-sporulating, spherical, facultative anaerobe from the human gastrointestinal tract (Additional file 1: Figure S1). It tolerates 6% NaCl in MRS broth. Growth occurs at 15–45°C and at pH 4–8 but optimally at 37°C. The colonies on the MRS agar were white, smooth, shiny, and circular with complete edges. Some carbohydrates, such as L-arabinose, D-ribose, D-xylose, D-galactose, D-glucose, D-fructose, D-mannose, N-acetylglucosamine, amygdalin, arbutin, salicin, D-cellobiose, D-maltose, D-trehalose, gentiobiose, and D-fucose, can be used as the sole carbon sources, whereas glycerol, erythritol, etc. cannot (Additional file 2: Table S1).

**Figure 1:**
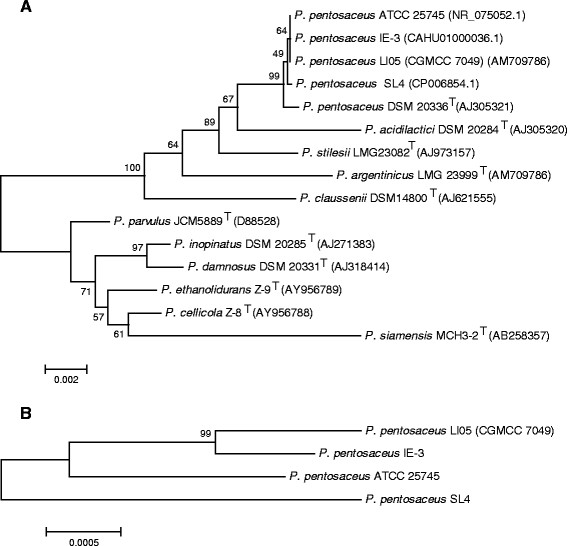
**The position of*P. pentosaceus*LI05 relative to the representative strains and the evolutionary relationships of the four strains of*P. pentosaceus*. A**. Phylogenetic tree highlighting the position of *P. pentosaceus* LI05 relative to the representative strains. The tree was constructed by the neighbor-joining method based on alignments of 16S rRNA gene sequences. Corresponding NCBI accession numbers are shown in parentheses. Numbers at the nodes indicate support values obtained from 1,000 bootstrap replications. **B**. Phylogenetic tree highlighting the evolutionary relationships of the four strains of *P. pentosaceus* based on concatenated nucleotide sequences of the *dnaA*, *dnaJ*, *dnaK*, *pheS*, *pryH*, *recA*, *recH*, *tuF*, *gryB* and *rplB* genes.

A neighbor-joining tree (Figure 1A) based on the 16S rRNA gene sequence of the strain LI05 shows the phylogenetic relationships between the species of the genus *Pediococcus.* This organism formed a distinct branch with *P. pentosaceus,* which was separate from those formed by other members of the genus *Pediococcus*. Sequence analyses of the *dnaA*, *dnaJ*, *dnaK*, *pheS*, *pryH*, *recA*, *recH*, *tuF*, *gryB* and *rplB* housekeeping genes were carried out for the definitive identifications of *P. pentosaceus* LI05, *P. pentosaceus* ATCC 25745, *P. pentosaceus* SL4 and *P. pentosaceus* IE-3. As shown in Figure 1B, the combination of the above housekeeping genes provided good phylogenetic resolution of the four strains. The *P. pentosaceus* strain IE-3 was the closest evolutionary relative of strain LI05.

### Genome properties

**Figure 2:**
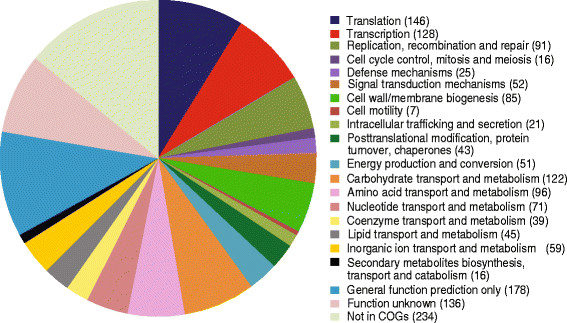
**The distribution of the genes associated with the 25 general COG functional categories in*P. pentosaceus*LI05.** The number of genes is shown in parentheses.

**Figure 3:**
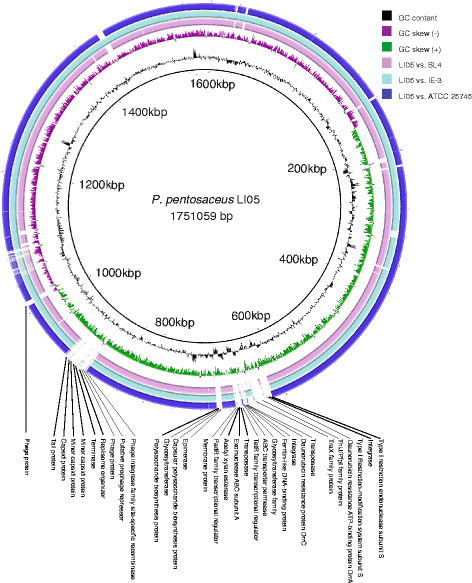
**BRIG BLAST analysis of the*P. pentosaceus*genomes using the genome of strain LI05 as the reference.** The strains and figure colors (from the center to the outside) represent LI05 vs. SL4 (pink), LI05 vs. IE-3 (light blue) and LI05 vs. ATCC 25745 (dark blue).

The genome of *P. pentosaceus* LI05 was sequenced by the Illumina method (see Methods). A total of 11.05 million 100-bp paired-end reads were generated, which provided over 500-fold coverage of the reference genome. High-quality reads with Q > 30 were assembled using *de novo* methods to obtain a draft genome of 1.75 Mbp with 8 contigs (the N50 of the assembled contigs was 34.3 Kb; the max length was 318 Kb). The G + C content of *P. pentosaceus* LI05 was 37.29%. For the main chromosome, 1,638 genes were predicted, 1,555 of which were protein-coding genes. A total of 1,321 protein-coding genes were assigned to putative functions, and the remainder were classified as hypothetical proteins. This genome contained 50 tRNAs and a complete 5S-23S-16S rRNA gene family. The properties and statistics of the genome are shown in Table 1 and Figure 2. As shown in Figure 3, the genome sequence of *P. pentosaceus* LI05 was highly conserved compared with those of *P. pentosaceus* ATCC 25745, *P. pentosaceus* SL4 and *P. pentosaceus* IE-3.

**Table 1:** Genomic nucleotide content and gene counts

**Attribute**	**Genome (total)**	
	**Value**	**% of total**^**a**^
Size (bp)	1,751,578	
G + C content (bp)	653,105	37.29
Coding region (bp)	1,457,159	83.19
Total genes^b^	1,638	
RNA genes	53	3.24
Protein-coding genes	1,555	94.93
Genes assigned to COGs	1,321	84.95
Genes with signal peptides	29	1.86
Genes with transmembrane helices	492	31.64

^a)^The total is based either on the size of the genome in base pairs or the total number of protein-coding genes in the annotated genome.

^b)^Also includes 35 other genes.

### Genome of *P. pentosaceus* LI05 exhibits probiotic properties

In a previous study, we have observed that *P. pentosaceus* LI05 is resistant to gastric acidity and bile compounds [7]. This was supported by the genomic data from this study, in which a gene encoding cholylglycine hydrolase, which is related to bile salt resistance, and genes encoding F0F1 ATP synthases, which are important for acid tolerance, were detected (Table 2). Additionally, six universal stress proteins (UspA), the chaperone protein DnaJ, the cofactor GrpE, which participates in the hyperosmotic and heat shock responses, the chaperone GroEL, which protects against environmental stresses, an enterocin A immunity family protein, an arsenate reductase, and methionine sulfoxide reductase A, which protects against oxidative stresses, were annotated. These representative stress resistance genes were highly conserved between *P. pentosaceus* LI05 and *P. pentosaceus* ATCC 25745, P. *pentosaceus* IE-3 or *P. pentosaceus* sL4, but most of them showed significant divergences from other species.

**Table 2:** **Comparison of important genes encoding stress resistance proteins in** ***P. pentosaceus*** **LI05,** ***P. pentosaceus*** **ATCC 25745,** ***P. pentosaceus*** **SL4 and** ***P. pentosaceus*** **IE-3**

**Characterization**	**Locus_tag**	**Size (bp)**	**Product description**	**Identity (BLASTx)**			**Max. identity to proteins of other species (BLASTp) (%)**
				**ATCC 25745 (%)**	**SL4 (%)**	**IE-3 (%)**	
Bile tolerance	BB06_RS0100955	1,026	Choloylglycine hydrolase family protein	99.05	99.25	100	69.50
Acid tolerance	BB06_RS0107040	1,518	F0F1 ATP synthase subunit alpha	99.74	99.60	95.39	92.46
	BB06_RS0107025	420	F0F1 ATP synthase subunit epsilon	99.76	99.29	99.76	84.89
	BB06_RS0107030	1,410	F0F1 ATP synthase subunit beta	99.86	99.86	100	96.16
	BB06_RS0107035	921	F0F1 ATP synthase subunit gamma	99.89	99.78	99.89	87.95
	BB06_RS0107050	522	F0F1 ATP synthase subunit B	99.81	99.81	100	90.53
	BB06_RS0107055	213	F0F1 ATP synthase subunit C	100	100	100	87.14
	BB06_RS0107060	717	F0F1 ATP synthase subunit A	100	99.86	99.86	96.22
Universal stress resistance	BB06_RS0103670	453	Universal stress protein UspA	99.12	99.34	99.12	68.42
	BB06_RS0106945	456	Universal stress protein UspA	100	100	100	90.67
	BB06_RS0101810	474	Universal stress protein UspA	99.37	99.79	99.79	70.70
	BB06_RS0100900	432	Universal stress protein UspA	99.54	99.07	99.77	99.30
	BB06_RS0101815	450	Universal stress protein UspA	99.33	99.11	99.11	93.24
	BB06_RS0102220	453	Universal stress protein UspA	99.78	99.78	100	91.21
Hyperosmotic and heat resistance	BB06_RS0104860	594	GrpE protein	98.32	98.82	99.83	72.60
	BB06_RS0104870	1,131	Molecular chaperone DnaJ	99.03	98.85	99.82	88.56
Environmental stress resistance	BB06_RS0102555	1,620	Molecular chaperone GroEL	100	99.26	100	94.81
Oxidative stress resistance	BB06_RS0105175	516	Methionine sulfoxide reductase A	99.22	99.22	99.61	81.87
Enterocin A resistance	BB06_RS0101010	279	Enterocin A Immunity family protein	99.64	-	100	38.89
Arsenate resistance	BB06_RS0105725	354	Arsenate reductase	99.15	98.87	100	83.76

Note: “-”, not detected.

The ability to adhere to gastrointestinal mucosa is an important property of most probiotics [21],[22]. Several proteins encoded by *P. pentosaceus* LI05 genes had predicted adhesive potentials (Table 3). For example, sortase attaches surface proteins, including enzymes, pilins and adhesion-mediating large surface glycoproteins, to cell walls. Other proteins included a pilin-like competence protein ComGC, elongation factor Tu (EF-Tu), an enolase capable of binding to host extracellular fibronectin and the pilus biosynthesis protein HicB. Abundant adhesion proteins encoded by the genomic regions were consistent with the strong adhesion properties of *P. pentosaceus* LI05. However, these proteins have also been predicted in the other tested strains. These findings may represent a possible reason for the extensive colonization of *P. pentosaceus* in the gut. However, the examinations of many more genes or proteins may be required to evaluate the adhesive abilities of probiotics.

**Table 3:** **Comparison of important genes encoding beneficial proteins in** ***P. pentosaceus*** **LI05,** ***P. pentosaceus*** **ATCC 25745,** ***P. pentosaceus*** **SL4 and** ***P. pentosaceus*** **IE-3**

**Characterization**	**Locus_tag**	**Size (bp)**	**Product description**	**Identity (BLASTx)**			**Max. identity to proteins of other species (BLASTp) (%)**
				**ATCC 25745 (%)**	**SL4 (%)**	**IE-3 (%)**	
Adhesion	BB06_RS0106620	306	Competence protein ComGC	99.67	99.67	100	76.47
	BB06_RS0106150	1,188	Elongation factor Tu	99.92	99.92	100	95.69
	BB06_RS0102755	1,323	Enolase	99.92	99.85	100	91.82
	BB06_RS0107170	330	Pilus biosynthesis protein HicB	100	100	100	60.91
	BB06_RS0108295	660	Sortase	99.70	99.24	99.85	76.71
	BB06_RS0102750	756	Triosephosphate isomerase	100	99.47	100	93.23
Antimicrobial	BB06_RS0101015	182	Prebacteriocin	100	-	-	96.30
	BB06_RS0105320		Lysin	92.84	91.02	-	86.70
	BB06_RS0100880	528	Colicin V production family protein	99.24	99.24	99.81	61.64
Biosynthesis of peptidoglycans	BB06_RS0102815	903	UDP-N-acetylenolpyruvoylglucosamine reductase	100	98.9	100	84.62
	BB06_RS0106375	1,368	UDP-N-acetylmuramoylalanine-D-glutamate ligase	99.05	98.90	100	74.23
	BB06_RS0107220	687	Peptidoglycan-binding protein LysM	99.85	99.71	99.85	67.54
	BB06_RS0100815	1,338	Peptidoglycan-binding protein	88.20	89.95	99.85	55.53
Riboflavin synthesis	BB06_RS0100520	471	Riboflavin synthase, beta subunit	98.09	97.74	60.30	61.43
	BB06_RS0100530	606	Riboflavin synthase, alpha subunit	98.84	97.85	100	61.14
	BB06_RS0100535	1,083	Riboflavin biosynthesis protein RibD	99.26	99.26	100	57.61
	BB06_RS0104850	945	Riboflavin biosynthesis protein RibF	97.46	99.26	100	74.84
	BB06_RS0105860	360	Riboflavin biosynthesis acetyltransferase (GNAT) family	100	99.72	100	84.14
Folate	BB06_RS0106895	1,275	Folylpolyglutamate synthase	99.45	99.53	-	59.52
	BB06_RS0105770	486	Dihydrofolate reductase	98.56	98.56	55.76	53.13
Gamma-aminobutyrate	BB06_RS0107660	1,452	Gamma-aminobutyrate permease	99.38	99.66	100	85.45
Biotin	BB06_RS0108625	560	Biotin biosynthesis protein BioY	99.47	99.47	100	55.19
Thiamine	BB06_RS0106910	1,188	Thiamine biosynthesis protein ThiI	99.67	99.67	100	88.64
	BB06_RS0107185	942	Thiamine biosynthesis protein ApbE	98.09	98.93	99.79	63.96

Note: “-”, no detection.

The *P. pentosaceus* LI05 genes also encoded three antimicrobials, which is consistent with the excellent antimicrobial ability of this strain. As shown in Table 3, genes encoding prebacteriocin were annotated in the genomes of both *P. pentosaceus* LI05 and *P. pentosaceus* ATCC 25745. Alternatively, the pedA gene (PCPN_1274) encoding pediocin PA-1 was detected in *P. pentosaceus* IE-3, but it was not identical to the prebacteriocin gene of *P. pentosaceus* LI05. Furthermore, genes encoding colicin V, which is a peptide antibiotic that kills sensitive cells by disrupting their membrane potentials [23], were found in these four *P. pentosaceus* strains. However, the colicin V discovered in strain L105 was different from that of the other spicies. Additionally, genes encoding lysin were detected in *P. pentosaceus* LI05 and *P. pentosaceus* ATCC 25745. As an antimicrobial agent, lysin is potentially immunogenic [24]. Therefore, *P. pentosaceus* LI05 can achieve “competitive exclusion” not only by limiting the surface area available but also by secreting antimicrobial substances.

In the genome of *P. pentosaceus* LI05, we also detected potentially beneficial properties that were not experimentally confirmed. This strain contained genes involved in the biosynthesis of not only important vitamins, such as riboflavin, folate, thiamine and biotin but also of functional factors, such as gamma-aminobutyrate (Table 3) [25]. In Gram-positive bacteria, peptidoglycan is one of the most important host immune regulators [26]. Although the genes and coding proteins related to the peptidoglycan pathway were conserved in the four strains of *P. pentosaceus*, they were not significantly similar to those of the other species. These findings will contribute to the elucidation of the mechanisms of immune regulation in *P. pentosaceus* LI05.

### Comparisons with other fully sequenced genomes

Fifty-three proteins encoded by *P. pentosaceus* LI05 genes were not detected or had sequence similarities of less than 50% in the comparative analysis with the three known food-borne strains, *P. pentosaceus* ATCC 25745, SL4 and IE-3. Among these proteins, 21 hypothetical proteins with no clear functions were not further analyzed; the other 32 proteins are listed in Table 4, demonstrated in Figure 3, and further discussed below.

**Table 4:** **Genes and their encoded proteins detected in** ***P. pentosaceus*** **LI05 with sequence similarities of less than 50% with sequences from both** ***P. pentosaceus*** **ATCC 25745 and** ***P. pentosaceus*** **SL4**

**Locus**	**Size (bp)**	**Predicted function**	**Best BLASTp hit**		**% Query cover**	**% Amino acid identity**
		**By BLASTp**	**Accession no.**	**Organism**		
BB06_RS0102945	1,110	Type I restriction endonuclease subunit S	WP_000072560.1	*Staphylococcus aureus*	100	49.34
BB06_RS0102950	912	Integrase	WP_006845852.1	*Weissella koreensis*	100	74.26
BB06_RS0102955	672	Type I restriction-modification system, specificity subunit S	WP_003595917.1	*Lactobacillus casei*	100	54.71
BB06_RS0102980	936	Daunorubicin resistance ATP-binding protein DrrA	YP_004841605.1	*Lactobacillus sanfranciscensis* TMW 1.1304	100	62.06
BB06_RS0102995	678	ThiJ/PfpI family protein	WP_010770374.1	*Enterococcus caccae*	99	71.42
BB06_RS0103035	633	TraX family protein	YP_006726711.1	*Lactobacillus buchneri* CD034	99	50.90
BB06_RS0103095	618	Transposase	YP_005004471.1	*Pediococcusclaussenii* ATCC BAA-344	100	98.05
BB06_RS0103100	2,268	Daunorubicin resistance protein DrrC	WP_003680292.1	*Lactobacillus coryniformis*	100	99.07
BB06_RS0103110	591	Integrase	WP_004906016.1	*Leuconostoc citreum*	99	97.96
BB06_RS0103120	468	Ferritin-like DNA-binding protein	BAN08201.1	*Lactobacillus plantarum 2025*	100	99.35
BB06_RS0103125	744	Glycosyltransferase family 2	WP_027822873.1	*Lactobacillus plantarum*	100	99.60
BB06_RS0103130	1,248	ABC transporter permease	WP_027822874.1	*Lactobacillus plantarum*	100	99.76
BB06_RS0103135	672	TetR family transcriptional regulator	WP_027822875.1	*Lactobacillus plantarum*	100	99.55
BB06_RS0103140	339	Transposase	WP_015474731.1	*Lactobacillus brevis*	95	99.07
BB06_RS0103155	1,308	Excinuclease ABC subunit A	WP_024862991.1	*Pediococcus acidilactic*i	99	72.51
BB06_RS0103175	859	Acetyl xylan esterase	WP_025478109.1	*Enterococcus saccharol*yticus	99	88.97
BB06_RS0103180	561	PadR family transcriptional regulator	WP_017552090.1	*Bacillus coagulans*	100	99.43
BB06_RS0103200	906	Membrane protein	024625654.1	*Lactobacillus fabifermentans*	99	45.18
BB06_RS0103305	930	Epimerase	WP_021357793.1	*Lactobacillus plantarum*	99	62.50
BB06_RS0103310	660	Capsular polysaccharide biosynthesis protein	WP_003680917.1	*Lactobacillus coryniformis*	93	60.68
BB06_RS0103315	846	Glycosyltransferase	WP_003638227.1	*Lactobacillus pentosus*	96	45.39
BB06_RS0103320	987	Polysaccharide biosynthesis protein	YP_004889104.1	*Lactobacillus plantarum* WCFS1	98	48.32
BB06_RS0104430	1,173	Phage integrase family site-specific recombinase	WP_004165738.1	*Pediococcus acidilactici DSM 20284*	99	75.19
BB06_RS0104450	398	Putative prophage repressor	WP_007289487.1	*Thermosinus carboxydivorans*	97	41.98
BB06_RS0104515	768	Phage protein	ERL43462.1	*Lactobacillus plantarum* JDM1	64	48
BB06_RS0104520	692	Replisome organizer	WP_004165758.1	*Pediococcus acidilactici*	99	66.09
BB06_RS0104600	1,374	Terminase	WP_002318686.1	*Enterococcus faecium*	100	60.18
BB06_RS0104605	1,548	Minor capsid protein	WP_022638369.1	*Lactobacillus plantarum*	98	58.75
BB06_RS0104610	1,134	Minor capsid protein	WP_016511174.1	*Lactobacillus plantarum*	99	49.47
BB06_RS0104645	462	Capsid protein	WP_002314916.1	*Enterococcus faecium*	92	72.03
BB06_RS0104660	5,241	Tail protein	WP_002820753.1	*Oenococcus oeni*	73	35.42
BB06_RS0105620	810	Phage protein	WP_012678830.1	*Streptococcus equi*	92	33.46

Five putative exopolysaccharide biosynthesis proteins were detected only in *P. pentosaceus* LI05, including an epimerase, a capsular polysaccharide biosynthesis protein, two glycosyltransferases (key enzymes for the biosyntheses of the exopolysaccharide repeating units) and a polysaccharide biosynthesis protein. Four of these enzymes need to be examined in further detail because they are not only potentially novel but also probably induce variations in the structures of their encoded polysaccharides that may have influenced adherence, biofilm formation and the nature of the immune response [27].

*P. pentosaceus* LI05 was characterized by three extra-environmental stress tolerance proteins, including a putative ferritin-like DNA-binding protein, which maintains a steady state of iron ions and responds to stresses, such as those involving temperature, humidity, and ionizing and redox processes [28], a putative PadR family transcriptional regulator, which functions against phenolic acid stress, and a putative ThiJ/PfpI family protein, which is involved in cellular protection against environmental stresses [29].

Fourteen proteins related to the intrusion of exogenous DNA were identified in *P. pentosaceus* LI05. One group was comprised of twelve prophage-related proteins, including a phage integrase family site-specific recombinase, two integrases, a putative prophage repressor, two phage proteins, a replisome organizer, a terminase, two minor capsid proteins, a capsid protein and a tail protein. It is not rare for bacteria to contain multiple prophages in their chromosomes, which then constitute a sizable proportion of their total chromosomal material [30]. Pathogenic, commensal, and symbiotic bacteria have been observed to play roles in a variety of bacterial adaptations in hosts [31]. Phage-related proteins were encoded by genes in each of the three food-borne strains. The other genes detected in the *P. pentosaceus* LI05 included two encoding bacterial DNA type I restriction endonucleases, which are involved in prokaryotic DNA restriction-modification mechanisms that protect the bacteria against invading foreign DNA [32].

Two putative doxorubicin-daunorubicin resistance proteins existed in *P. pentosaceus* LI05. One ORF encoded DrrA, which is part of the ABC transporter complex DrrAB. The other ORF encoded DrrC, which is part of the ABC transporter permease protein. This finding partially reflects the complex interactions between drugs and gut-associated microbes [33]. Both daunorubicin and doxorubicin are antitumor drugs and are thus not suitable for antibacterial applications. Therefore, these two genes will not affect the control of *P. pentosaceus* LI05.

Additionally, there were eight extra putative multifunctional proteins in *P. pentosaceus* LI05. These included a TetR family transcriptional regulator, an ABC transporter permease, an exonuclease ABC subunit A, a transposase, an acetyl xylan esterase, a PadR family transcriptional regulator, a membrane protein and a TraX family protein.

## Conclusions

Strains of *P. pentosaceus* are frequently identified in food and in the human gastrointestinal tract and are known to reduce inflammation, encephalopathy, obesity and fatty liver in animals. Therefore, it is imperative to study the probiotic ability of this organism. Future studies will focus on delineating the interactions between the host and *P. pentosaceus*. The genome sequences of *P. pentosaceus* LI05 isolated from the human gastrointestinal tract allow for a deeper understanding of its probiotic abilities, facilitating the future development of drugs for microbiota-related diseases.

### Availability of supporting data

The whole-genome sequencing project of *P. pentosaceus* LI05 has been submitted to GenBank under the project accession number PRJNA237570. The project version entailing the draft assembly described herein has been deposited under the accession number JDVW00000000.

## Competing interests

The authors declare no competing interests.

## Authors’ contributions

L-JL designed the study, interpreted the results and edited the manuscript. L-XL and Y-DL conducted the Illumina sequencing, performed the assemblies, analyzed the genome, and performed the annotations. X-JH provided advice related to the outbreak and strain features, characterized the strain and maintained it in pure cultures. H-YS contributed to the microbiology of the strain and prepared high-molecular-weight DNA for the genome sequencing. All authors read and approved the manuscript prior to submission.

## Additional files

## Supplementary Material

Additional file 1: Figure S1. Scanning electron micrograph (A) and transmission electron micrograph (B) of *P. pentosaceus* LI05. Click here for file

Additional file 2: Table S1. Ability of *P. pentosaceus* LI05 to grow using specific carbohydrates. Click here for file
