# Supplementary figures and images for: Whole-genome sequence assembly of Pediococcus pentosaceus LI05 (CGMCC 7049) from the human gastrointestinal tract and comparative analysis with representative sequences from three food-borne strains
Source: Gut Pathog. 2014 Aug 30;6:36. doi: 10.1186/s13099-014-0036-y (PMC4209512; doi:10.1186/s13099-014-0036-y)

**A****1  $\mu\text{m}$** 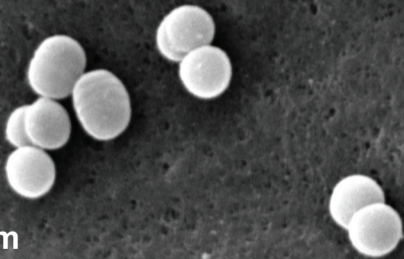**B****1  $\mu\text{m}$** 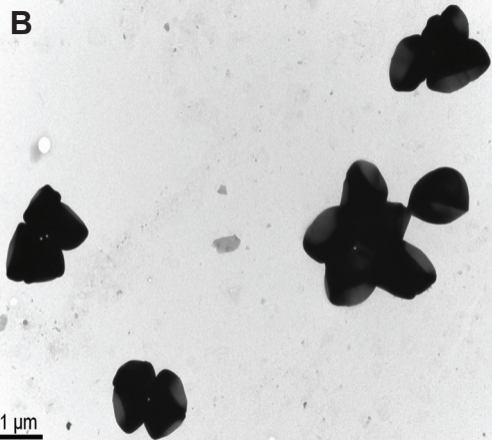

Supplement: Additional file 1: Figure S1. — Scanning electron micrograph (A) and transmission electron micrograph (B) of P. pentosaceus LI05. [file s13099-014-0036-y-S1.pdf]
