# Supplementary material for: Whole-genome sequence assembly of Pediococcus pentosaceus LI05 (CGMCC 7049) from the human gastrointestinal tract and comparative analysis with representative sequences from three food-borne strains
Source: Gut Pathog. 2014 Aug 30;6:36. doi: 10.1186/s13099-014-0036-y (PMC4209512; doi:10.1186/s13099-014-0036-y)
Supplement: Additional file 2: Table S1. — Ability of P. pentosaceus LI05 to grow using specific carbohydrates. [file s13099-014-0036-y-S2.doc]

Additional file 1: Table S1. Ability of *P. pentosaceus* LI05 to grow using specific carbohydrates

| Carbohydrate name | Growth | Carbohydrate name | Growth | Carbohydrate name | Growth |
| --- | --- | --- | --- | --- | --- |
| Control | - | Glycerol | - | D-saccharose | - |
| L-arabinose | + | Erythritol | - | Inulin | - |
| D-ribose | + | D-arabinose | - | D-melezitose | - |
| D-xylose | + | L-xylose | - | D-raffinose | - |
| D-galactose | + | Methyl-βD-xylopyranoside | - | Amidon | - |
| D-glucose | + | L-sorbose | - | Glycogen | - |
| D-fructose | + | L-rhamnose | - | Xylitol | - |
| D-mannose | + | Dulcitol | - | D-turanose | - |
| N-acetylglucosamine | + | Inositol | - | D-lyxose | - |
| Amygdalin | + | D-mannitol | - | D-tagatose | - |
| Arbutin | + | D-sorbitol | - | L-fucose | - |
| Salicin | + | D-adonitol | - | D-arabitol | - |
| D-cellobiose | + | Methyl-αd-mannopyranoside | - | L-arabitol | - |
| D-Maltose | + | Methyl-αd-glucopyranoside | - | Potassium gluconate | - |
| D-trehalose | + | Esculin ferric citrate | - | Potassium 2-ketogluconate | - |
| Gentiobiose | + | D-lactose | - | Potassium 5-ketogluconate | - |
| D-fucose | + | D-melibiose | - |  |  |

Note: “+”, growth; “-”, no growth.
